# Estimating the contribution of different age strata to vaccine serotype pneumococcal transmission in the pre vaccine era: a modelling study

**DOI:** 10.1186/s12916-020-01601-1

**Published:** 2020-06-10

**Authors:** Stefan Flasche, Marc Lipsitch, John Ojal, Amy Pinsent

**Affiliations:** 1 Centre for Mathematical Modelling of Infectious Diseases, Department of Infectious Disease Epidemiology, London School of Hygiene & Tropical Medicine, Keppel Street, London, UK; 2 Center for Communicable Disease Dynamics, Department of Epidemiology and Department of Immunology and Infectious Diseases, Harvard School of Public Health, 677 Huntington Avenue, Boston, MA USA

**Keywords:** *S. pneumoniae*, Transmission, Vaccination, Schedules, Modelling

## Abstract

**Background:**

Herd protection through interruption of transmission has contributed greatly to the impact of pneumococcal conjugate vaccines (PCVs) and may enable the use of cost-saving reduced dose schedules. To aid PCV age targeting to achieve herd protection, we estimated which population age groups contribute most to vaccine serotype (VT) pneumococcal transmission.

**Methods:**

We used transmission dynamic models to mirror pre-PCV epidemiology in England and Wales, Finland, Kilifi in Kenya and Nha Trang in Vietnam where data on carriage prevalence in infants, pre-school and school-aged children and adults as well as social contact patterns was available. We used Markov Chain Monte Carlo methods to fit the models and then extracted the per capita and population-based contribution of different age groups to VT transmission.

**Results:**

We estimated that in all settings, < 1-year-old infants cause very frequent secondary vaccine type pneumococcal infections per capita. However, 1–5-year-old children have the much higher contribution to the force of infection at 51% (28, 73), 40% (27, 59), 37% (28, 48) and 67% (41, 86) of the total infection pressure in E&W, Finland, Kilifi and Nha Trang, respectively. Unlike the other settings, school-aged children in Kilifi were the dominant source for VT infections with 42% (29, 54) of all infections caused. Similarly, we estimated that the main source of VT infections in infants are pre-school children and that in Kilifi 39% (28, 51) of VT infant infections stem from school-aged children whereas this was below 15% in the other settings.

**Conclusion:**

Vaccine protection of pre-school children is key for PCV herd immunity. However, in high transmission settings, school-aged children may substantially contribute to transmission and likely have waned much of their PCV protection under currently recommended schedules.

## Background

*Streptococcus pneumoniae* is a common coloniser of the human nasopharynx and a major cause of acute otitis media, pneumonia, sepsis and meningitis worldwide [1, 2]. Pneumococcal conjugate vaccine (PCV) introduction to infants [3] has helped mitigate that burden [4–6]; however, the long-term success of routine vaccination is challenged by serotype replacement [7, 8].

Herd protection has been an integral part of the success of PCVs [9, 10]. In particular, in high-income countries, reduced acquisition of infections with vaccine-targeted serotypes in immunised infants and toddlers has almost eliminated their circulation, and hence associated disease, in the whole population. This has supported the notion that the impact of a mature programme may be sustained by a reduced dose schedule that retains the impact of the programme by maintaining herd effects albeit at the potential cost of reduced direct protection during infancy [11]. Indeed, it has subsequently been shown that a booster dose schedule with only a single priming dose induces largely non-inferior post booster IgG responses, when compared to the currently WHO recommended 2 priming and booster dose schedule, indicating similar post booster protection and hence the potential to sustain herd effects [12]. In 2017/2018, the UK’s Joint Committee on Vaccination and Immunisation reviewed this evidence and recommended the switch to a reduced 1p+1 PCV dosing schedule for the UK [13–15]. There is, however, limited information on the extent to which decreased PCV protection in infancy with a reduced dose schedule may lead to an increase in vaccine serotype circulation in the community, particularly in infants. In a reduced dose schedule, infants may be exposed substantially more frequent to vaccine serotypes, which in combination with their likely reduced direct protection may lead to a resurgent of vaccine serotype pneumococcal disease in this age group. Quantifying the contribution of infants and other age groups to overall pneumococcal transmission dynamics (i.e. the force of infection) would aid an assessment of such risk.

Similar to routine vaccination programmes, PCV use in campaigns, i.e. the immunisation of a number of birth cohorts within a confined time window, can substantially benefit from induced herd immunity in order to optimise their efficiency. Campaigns are used either to accelerate the impact of PCV at introduction [16, 17] or to implement rapid vaccination in settings, including humanitarian crises, where routine infant immunisation is interrupted or unfeasible [18]. The optimal age targeting of such campaigns depends on a combination of factors including maximising direct protection, targeting core transmission groups and accommodating programmatic considerations. In many humanitarian crises, frequent population movement, security concerns or accessibility and cold chain problems would complicate a multi-dose PCV campaign approach [19]. Hence, PCV campaign design should include maximising the level of herd immunity achieved in order to protect the most vulnerable age groups who will only receive partial protection from a single dose. There is, however, limited information on which age groups are the main drivers of pneumococcal transmission within the population and hence warrant targeting to help interrupt transmission [20].

Evidence on pneumococcal transmission pathways stems largely either from assessing risk factors for colonisation in cross-sectional carriage surveys [21–23] or from inferring pneumococcal transmission based on timing and geographical relatedness of serotype-specific carriage events, observed typically in household-based longitudinal studies [24–27]. These suggest that household exposure and child care attendance are important routes for pneumococcal transmission, that transmission is driven mainly by young children rather than adults and that infants are more likely to acquire carriage from siblings than from their parents. Further, the near elimination of vaccine serotypes within a few years after introduction of PCV programmes in infants and toddlers suggests a disproportional contribution of young children in pneumococcal transmission [10]. However, none of the studies published to date have tried to quantify the proportional contribution of different age strata across the population to pneumococcal transmission, partly because of the difficulty in inferring transmission events. We aim to address this key evidence gap.

Using mathematical modelling, we combine information on pneumococcal carriage, local demographics and human contact patterns from four epidemiologically distinct contexts to estimate local age-specific pneumococcal transmission patterns at the population level.

## Methods

### Data

We reviewed the literature for countries that have reported both (i) pre-PCV introduction nasopharyngeal carriage among healthy young children (< 5 years), older children (5–17 years) and adults (18+ years) and (ii) a survey of human contact patterns relevant to the spread of pneumococci. From a systematic review of pre-PCV carriage studies globally (Le Polain de Waroux O: Global landscape of Streptococcus pneumoniae serotypes colonising healthy individuals worldwide; a systematic review and metaanalysis, Pers. Commun. 2016) [28], we identified 14 countries with studies that reported carriage prevalence among healthy individuals for each of the three age groups (Chile, Finland, Gabon, The Gambia, Israel, Kenya, Malawi, Nigeria, Papua New Guinea, South Africa, Sweden, UK, USA and Vietnam). Of those, we identified England, Finland, Kilifi in Kenya and Nha Trang in Vietnam to also have collected data on social contact patterns [29–35]. For England, Kilifi and Finland, age-stratified estimates of clearance rates for pneumococcal carriage episodes were also available [34, 36–38]. In the absence of local data for Nha Trang, we assumed clearance rates to be similar to those observed in England and Wales because of the similarity in carriage prevalence in these settings. Data on pneumococcal carriage prevalence and serotype distribution were extracted as finely age-stratified as possible but to capture at least infants, young children, school-aged children and adults separately. Most of the data was collected during 2000 to 2010 with all data on the epidemiology of pneumococcus being collected before PCV introduction (see Tables 1 and 2). We assume that during that time and before the introduction of PCV, the epidemiology of pneumococcus in the respective settings as well as the population demographic and contact patterns remain unchanged during that time.

**Table 1:** Year of data collection for model parameters and references to the relevant studies in brackets thereafter

	England and Wales	Kilifi	Nha Trang	Finland
Demographics	2001/2002 [39]	2009 [40]	2006 [41]	2000 [42]
Physical contacts	2005/2006 [29]	2011 [31]	2010 [35]	2005/2006 [29]
Carriage	2001/2002 [30]	2009 [32]	2008 [43]	1994–2002 [34]
Clearance rates	2001/2002 [36]	2006–2008 [37]	See E&W	2001/2002 [34, 38]
PCV product—introduction	PCV7—2006 PCV13—2010	PCV10—2011	No routine PCV use	PCV10—2010

**Table 2:** Key model input parameters for the four settings, stratified by vaccine serotypes (VT) and non-vaccine serotypes (NVT) where appropiate

	Age	Population	Carriage prevalence		Sample size	Clearance rate	
			VT	NVT		VT	NVT
**E&W**	< 1 year	629,200	0.43	0.12	51	0.014	0.014
	1–5 years	3,009,200	0.25	0.15	138	0.029	0.029
	6–14 years	5,999,000	0.08	0.10	52	0.056	0.056
	15–20 years	4,132,900	0.00	0.13	8	0.057	0.057
	21–49 years	21,368,700	0.00	0.02	232	0.059	0.059
	50+	17,905,900	0.00	0.00	2	0.059	0.059
**Kilifi**	< 1 year	9617	0.41	0.46	39	0.009	0.012
	1–5 years	45,170	0.34	0.44	127	0.020	0.023
	6–14 years	68,547	0.15	0.39	82	0.049	0.050
	15–20 years	33,289	0.14	0.25	56	0.049	0.050
	21–49 years	72,143	0.07	0.22	97	0.049	0.050
	50+	24,214	0.04	0.20	104	0.049	0.050
**Finland**	< 1 year	56,748	0.12	0.05	400	0.022	0.022
	1–5 years	284,828	0.24	0.10	412	0.022	0.022
	6–17 years	764,192	0.08	0.03	86	0.033	0.033
	18+	4,130,843	0.02	0.01	263	0.033	0.033
**Nha Trang**	< 1 year	2094	0.27	0.07	41	0.014	0.014
	1–5 years	15,239	0.25	0.20	63	0.029	0.029
	6–17 years	40,324	0.02	0.09	55	0.056	0.056
	18–49 years	115,538	0.01	0.01	262	0.059	0.059
	50+	37,544	0.01	0.01	98	0.059	0.059

We stratify carriage prevalence by vaccine and non-vaccine type carriage. Based on the current national programmes in the respective settings, we define vaccine types as the serotypes targeted by PCV13 for England and Wales (E&W) and as the serotypes targeted by PCV10 including cross protection against type 6A for Finland and Kilifi [18]. PCV is currently not included in the routine infant immunisation schedule in Vietnam. In line with an ongoing cluster-randomised trial in Nha Trang, we chose to define vaccine types in this setting as those serotypes targeted by PCV10 and serotype 6A [44].

Contact studies nested into carriage surveys in both Uganda [22] and Fiji [45] have recently shown that physical (i.e. skin to skin contact) and not conversational contacts are risk factor for pneumococcal carriage. This provides evidence that physical contacts are a better proxy for contacts relevant for pneumococcal transmission than the often used conversational contacts. Hence, we used reported physical contacts (Table 1) to populate setting-specific mixing matrices using established methods [46].

### Transmission models

For each of the four settings, we used a deterministic age-structured model that simulates the transmission dynamics of pneumococcal carriage for the pooled vaccine serotype and the non-vaccine serotype groups, similar to previous approaches [47, 48]. We assume a realistic but static population demographic (Tables 1 and 2). The study population is divided into susceptible (*S*), carriers of vaccine serotypes VT (*V*), carriers of non-vaccine serotypes NVT (*N*) and carriers of both VT and NVT at the same time (*B*). Due to the pooling of serotypes, no capsular-specific immunity following infection was included; however, carriers of either serotype group were unable to acquire additional infection with that group while carrying and were at reduced susceptibility for acquisition of carriage with the other serotype group (i.e. competition). Capsular non-specific protection was assumed to accrue with age to resemble a steady state proxy for the accrual of immunity with repeated exposure, and hence, we estimated the susceptibility to infection given potentially infectious contact for each setting and each of infants, pre-school children, school children and adults independently. The set of ordinary differential equations that govern the model read:

$$ {\displaystyle \begin{array}{c}{S}^{\prime }=-\left({\lambda}_V+{\lambda}_N\right)S+{\nu}_VV+{\nu}_NN\\ {}{V}^{\prime }={\lambda}_VS-\zeta {\lambda}_NV-{\nu}_VV+{\nu}_NB\\ {}\begin{array}{c}{N}^{\prime }={\lambda}_NS-\zeta {\lambda}_VN-{\nu}_NN+{\nu}_VB\\ {}{B}^{\prime }=\zeta \left({\lambda}_VN+{\lambda}_NV\right)-\left({\nu}_V+{\nu}_N\right)B.\end{array}\end{array}} $$

$$ {\displaystyle \begin{array}{c}{\lambda}_V={\beta}_V\ \left(V+B\right)\\ {}\ {\lambda}_N={\beta}_N\ \left(N+B.\right)\end{array}} $$

Age and time dependencies were omitted from the equations to enhance readability. *λ* is the serotype-group-specific force of infection. *ζ*, the reduction in acquisition rate if carrying, was assumed to be 0.1 in accordance with estimates from model fitting to post-PCV introduction changes [16, 48, 49]. *ν* represents the serotype-group- and age-group-specific clearance rates. *β* represents the age-group-specific effective contact rates who are a multiplication of the observed contact rates with the age-specific susceptibility to infection *θ*. This model includes a number of drastic simplifications of pneumococcal epidemiology, including that it artificially promotes the stable serotype (-group) coexistence [50]. All models stratify the population into at least infants, pre-school children, school-aged children and adults (exact age strata in Table 2).

### Parameter inference

Country-specific models were run to reach steady state to replicate VT and NVT carriage prevalence pre-PCV introduction, each model was fitted independently using a Bayesian inference framework that samples parameter posterior distributions using adaptive Markov Chain Monte Carlo (MCMC) [51, 52]. The multinomial likelihood of observing the age-dependent prevalence estimates for non-carriers, VT carriers and NVT carriers given the model predictions was maximised by estimating the age-specific (for < 1 year, pre-school children, school children and adults) probability for transmission given a potentially infectious contact. It was assumed that in the event of carriage of both VT and NVT simultaneously either VT or NVT would be identified at equal probability [53]. In each setting, the probability for transmission given a potentially infectious contact was estimated for the four age groups: infants, pre-school children, school children and adults, each stratified by VT and NVT (*θ*_1_, …, *θ*_8_). Hence, for each setting, the likelihood of *θ* given the observed data *x* is given by:

$$ L\left(\theta |x\right)\propto \prod \limits_{a=1}^n{p}_{Va}^{x_{Va}}\left(\theta \right)\ {p}_{Na}^{x_{Na}}\left(\theta \right)\ {p}_{Sa}^{x_{Sa}}\left(\theta \right) $$

where the age dependent *p*_*Va*_, *p*_*Na*_, *p*_*Sa*_ and *x*_*Va*_, *x*_*Na*_, *x*_*Sa*_ represent the prevalence of carriage in VT, NVT and non-carriers in the steady state of the model and the number of observed carriage events in the data, respectively, stratified into *n* age groups. Individuals in the model who are dual carriers were assumed to be observed as VT type carriers as dual carriage was not ascertained in the data sets used. Age dependencies of *p*_*i*_ and *x*_*i*_ were omitted for readability.

To encompass the uncertainty of the observed mixing patterns, particularly in infants, in the model, we bootstrapped the participants included in the mixing matrix calculations [54]. Starting with the pool of all unique participants to calculate the mixing matrix, every iteration of the MCMC, 5 contact survey participants (about 1% of the overall sample) were randomly selected, excluded, and replaced by a set of 5 participants that were sampled with replacement from the original pool of participants. For each MCMC iteration, the mixing matrix was updated according to the current pool of mixing survey participants. Replacing about 1% of participants per iteration was found to allow good exploration of the mixing matrix parameter space. MCMC convergence was assessed through Gweke criterion, and parameter correlation was ascertained visually.

### Sensitivity analyses

In principle, both the susceptibility to pneumococcal carriage acquisition and the transmissibility when carrying can change with age. However, only one of them can be fitted here because of identifiability limitations given the available data. We chose to fit the susceptibility to carriage acquisition given a potentially infectious contact and assumed that the probability of transmitting given a potentially infectious contact would be the same across all age groups. This was done in order to simulate a combination of general and pneumococcal exposure related maturation of the immune system with age, consistent with previously published work [34, 48, 49, 55]. For sensitivity analyses, however, we also explored fitting the transmissibility and assuming the susceptibility was the same across all age groups.

We also included additional sensitivity analyses to test the robustness of the results: (i) assuming Kilifi-like waning for the Nha Trang model, (ii) assuming all social contacts are relevant for the spread of pneumococci and (iii) the between serotype group competition is rather lower (*c* = 0.3) than assumed at base case.

All data analyses were conducted in R 3.6.1 [56]. The project code is available under https://github.com/StefanFlasche/Pneumo_Trans_Inf.

## Results

**Fig. 1:**
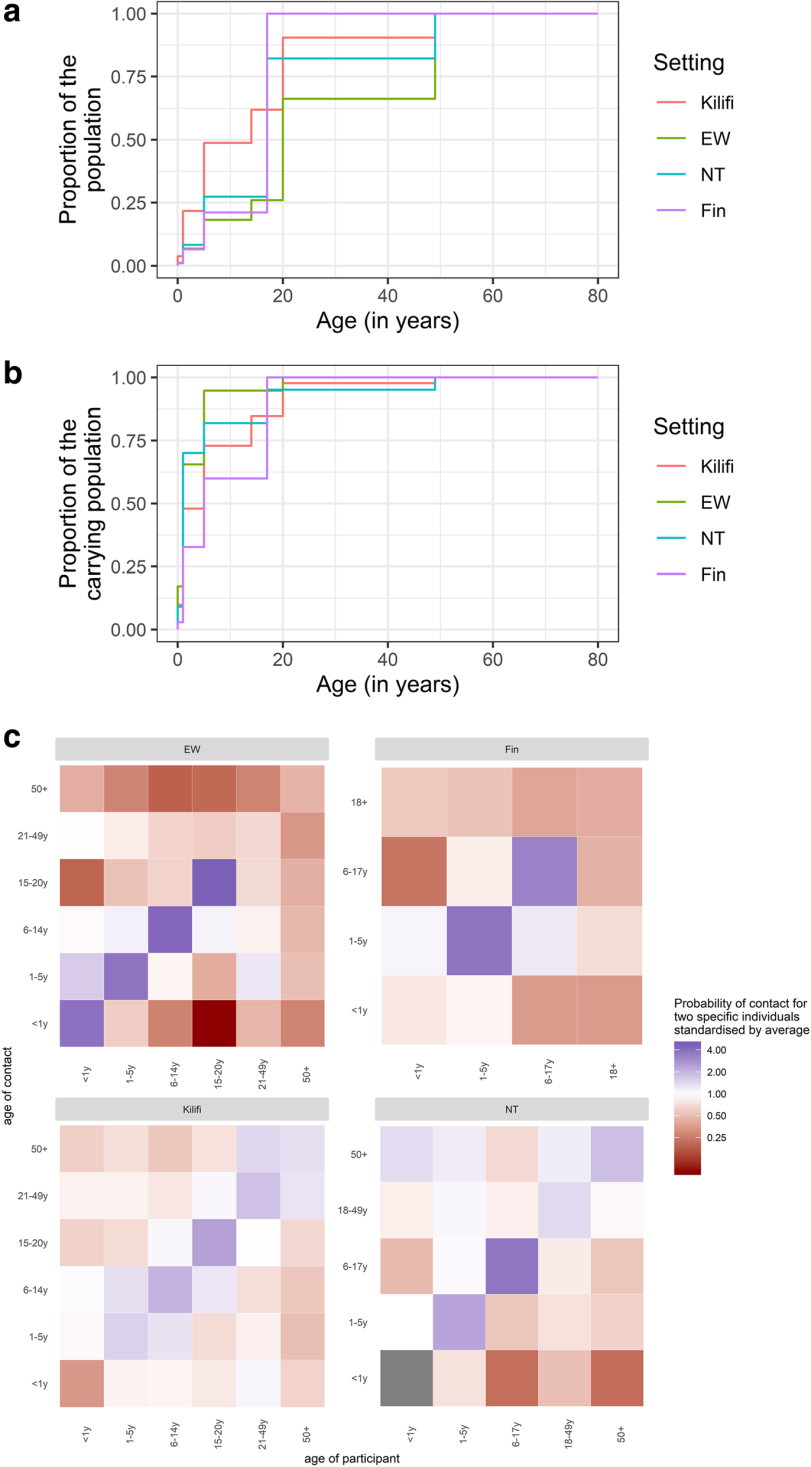
Demographic and epidemiological characteristic of the four study sites Kilifi, England and Wales (EW), Nha Trang (NT) and Finland (Fin). The top panel shows the cumulative age distributions within each setting. The middle panel shows the cumulative distribution of carriers in the population. The bottom panel compares the country-specific probabilities of physical contact of two specific individuals each standardised by the respective country specific average. They grey-coloured tiles indicate no reported contacts

Patterns of pneumococcal carriage in the pre-PCV era were qualitatively similar across study sites with highest carriage prevalence of vaccine and non-vaccine serotypes in young children, but generally declining with increasing age. In contrast to the other sites, carriage prevalence in adults in Kilifi remained comparatively high among adults with 4% VT and 20% NVT carriage prevalence among adults older than 50 years of age. Yet, the estimated proportion of all pneumococcal carriers who are adults was not higher in Kilifi than in the other settings because the high carriage rates in adults were counter-balanced by the relatively small contribution of adults to the population pyramid in Kilifi (Fig. 1).

All contact studies were well powered with 947, 890, 568 and 2002 participants from E&W, Finland, Kilifi and Nha Trang, respectively. However, with the exception of Kilifi, the surveys included less than 18 participating infants. In all settings, the reported frequency of physical contacts was highest within the age groups of pre-school and school-aged children. In Kilifi, less variance in contact frequency across ages was observed. While no infant-to-infant contact was reported in Nha Trang and little in Kilifi, respective contact rates were among the highest across age groups in E&W.

**Fig. 2:**
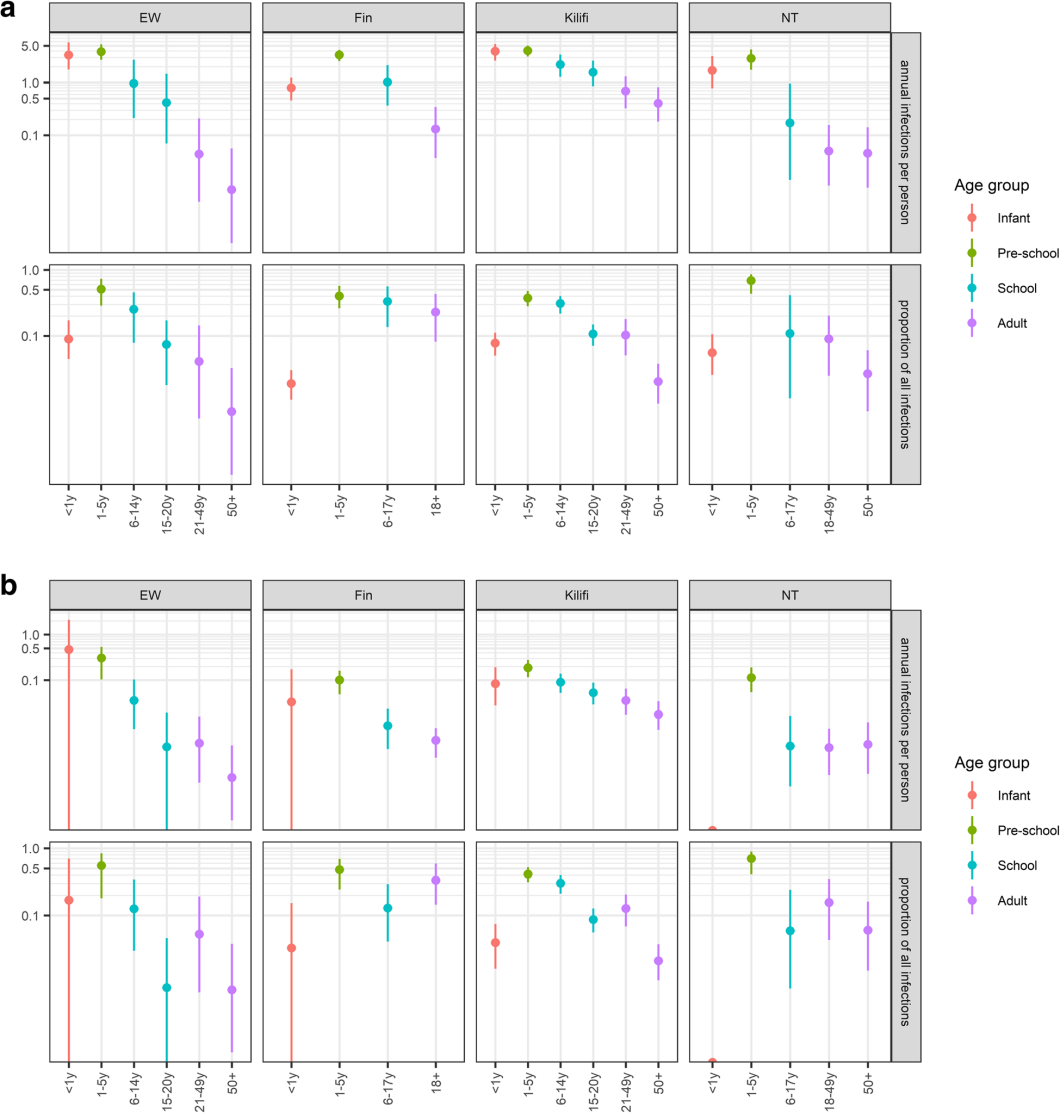
Attribution of vaccine serotype pneumococcal infections to population age strata that cause those infections for the four study sites Kilifi, England and Wales (EW), Nha Trang (NT) and Finland (Fin). Top panel: The top row illustrates the per capita annual number of infections in the total population that can be attributed to the respective population age strata (*x*-axis). Similarly, the bottom row illustrates the proportion of infections in the total population that can be attributed to respective age strata (*x*-axis). Bottom panel: The top row illustrates the per capita annual number of infections among infants that can be attributed to the respective population age strata (*x*-axis). Similarly, the bottom row illustrates the proportion of infections among infants that can be attributed to respective age strata (*x*-axis)

**Fig. 3:**
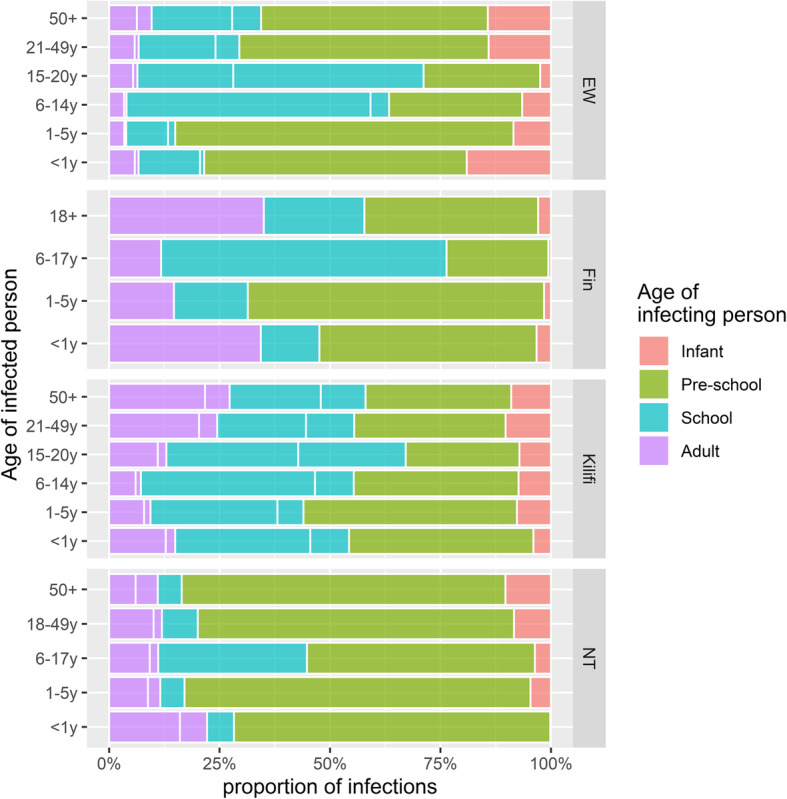
Contribution of different population age strata (grouped into four colours) to the vaccine type infection pressure in each age stratum (*y*-axis), stratified by settings Kilifi, England and Wales (EW), Nha Trang (NT) and Finland (Fin). For example, the dominance of green in a specific stacked bar indicates that among the vaccine type pneumococcal infections in this age group and this setting most stem from pre-school-aged children

In all settings, the model captured the data well (Additional file 1: Fig. S1 & S2). We estimate that per year an average person in E&W, Finland, Kilifi and Nha Trang infects 0.4 (95% quantile of the posterior (CrI), 0.3, 0.7), 0.5 (95% CrI, 0.3, 0.7), 1.9 (95% CrI, 1.5, 2.5) and 0.4 (95% CrI, 0.2, 0.6) individuals with pneumococcal vaccine serotypes. However, the average person in Kilifi is 15 years old and therefore half as old as an average person in the other settings. In all settings, infants exert a disproportionally high per capita infection pressure on the community. We estimate that the average infant from E&W, Finland, Kilifi and Nha Trang infects 3.3 (95% CrI, 1.7, 5.8), 0.8 (95% CrI, 0.5, 1.2), 3.9 (95% CrI, 2.6, 5.4) and 1.8 (95% CrI, 0.9, 3.2) persons during their first year of life (Fig. 2a). However, in each setting, this represents less than 10% of the overall infection pressure. Pre-school and school-aged children contribute the majority of infections with 86% (95% CrI, 71, 94), 76% (95% CrI, 56, 90), 80% (95% CrI, 70, 87) and 81% (95% CrI, 59, 93) in E&W, Finland, Kilifi and Nha Trang (Fig. 3). While among those children in E&W, Finland and Nha Trang, the biggest contribution to the infection pressure originates in pre-school children, in Kilifi the group of school-aged children contributes more to the infection pressure than pre-schoolers and we estimate that 42% (95% CrI, 29, 54) of all vaccine serotype pneumococcal acquisitions in the pre vaccine era in Kilifi are transmitted from school-aged children.

We estimate that an average person in E&W, Finland, Kilifi and Nha Trang infects 0.03 (95% CrI, 0.2, 0.4), 0.01 (95% CrI, 0.01, 0.02), 0.08 (95% CrI, 0.05, 0.11), 0.02 (95% CrI, 0.01, 0.03) infants with VTs every year. In most settings, pre-school children exhibit the highest per capita contribution to the infection pressure on infants. We find that a pre-school child on average infects 0.30 (95% CrI, 0.11–0.53), 0.10 (95% CrI, 0.05–0.16), 0.19 (95% CrI, 0.12–0.28) and 0.17 (95% CrI, 0.09–0.28) infants per year in E&W, Finland, Kilifi and Nha Trang (Fig. 2b). Similarly, the proportion of infant infections originating from pre-school and school-aged children is 72% (95% CrI, 26, 98), 63% (95% CrI, 37, 84), 81% (95% CrI, 72, 88) and 80% (95% CrI, 55, 95), while the contribution of other infants as the source of infection was 18% (0, 70) in E&W and below 4% in the other settings (Fig. 3).

In our sensitivity analysis, we assumed that transmissibility rather than susceptibility varies with age. Overall, the results were qualitatively similar in that pre-school children were estimated to play a key role in overall pneumococcal transmission dynamics and in the transmission to infants, with the exception of E&W where infants were attributed a much larger share of the overall force of infection (Figs. S4 & S5). Furthermore, we showed that our results are not sensitive to assuming (i) Kilifi-like clearance rates of pneumococcal carriage in Nha Trang (Additional file 1: Fig. S6), (ii) all social contacts rather than only physical contacts are relevant to the transmission for pneumococci (Additional file 1: Fig. S7) or (iii) less between serotype group competition (Additional file 1: Fig. S8).

## Discussion

In this study, we combine information on population demographics, age-stratified pre-PCV pneumococcal carriage prevalence and social contact patterns to estimate the pneumococcal transmission dynamics in four settings which represent low, middle and high income. Specifically, we quantify the contribution of different age strata to the infection pressure in the whole population and to infants particularly. We find qualitatively similar patterns in that the largest percentage of transmission events, i.e. more than 75% in all settings, are likely to be coming from pre-school and school-aged children. While infants have the highest per capita transmission rates, their contribution to the overall burden of pneumococcal infections was estimated to be limited. This is likely to be because only a small proportion of all pneumococcal carriers fall into that age range in combination with a relatively low number of transmission relevant social contacts (i.e. physical contacts) of infants, although there is only limited data on infant contacts. We estimate that infants acquire the majority of their pneumococcal infections from pre-school children; however, in Kilifi where carriage rates remained relatively high until early adulthood, many infant infections are likely to be acquired from older children as well.

While a number of pneumococcal transmission studies have identified risk factors for transmission, including pneumococcal exposure from the household [23, 27] or day care centre attendance [21], few have investigated the drivers of pneumococcal transmission in the overall population. Weinberger et al. use Israeli surveillance of IPD, carriage and vaccine uptake shortly after PCV introduction to infer that the observed herd effect in adults was temporally associated with high vaccination coverage in 36–59-months old children [57]. Althouse et al. used a longitudinal household carriage study to infer pneumococcal transmission in the respective Native American communities by epidemiologically linking carriage episodes hierarchically to either same serotype carriage observed in the household, the community or importation [24]. They similarly found that toddlers and older children drive pneumococcal transmission and that infants are not a key source of transmission in the community. While our results agree with these findings, they offer additional age-granularity, depending on the available stratification of the contact and carriage data, that helps with a better understanding on optimal PCV age targeting for inducing herd effects in a population and the advantage of relying only on cross-sectional data on pneumococcal carriage and social contacts, which are much easier to collect than longitudinal carriage studies or routine surveillance.

We find that despite a higher estimated per capita transmission rate than other age groups, infants do not contribute substantially to the overall force of pneumococcal infection. This supports that leaving infants with inferior protection under a reduced primary dose schedule [11, 12] may not substantially impair population herd effects that are mainly maintained through protection from the booster dose in toddlers and young children. Furthermore, we find that infants receive their vaccine type infections predominantly from toddlers and older pre-school children who will retain direct protection from PCV in a 1p+1 schedule; hence, infants should continue to benefit indirectly from PCV vaccination, although, in Kilifi, we find that substantial part of VT infant infections stemmed from school-aged children and hence may pose a risk to a reduced dose strategy in such high transmission settings. While our findings are based on pre-PCV epidemiology, it is likely that the kinetics of vaccine serotype transmission change with PCV use toward higher dominance of age groups not directly protected by the infant programme [45].

In Kilifi, high pneumococcal carriage prevalence extends into adulthood, similar to other high burden settings in Africa [58, 59]. As a result, we estimated that the per capita rate of pneumococcal transmission among Kilifi adults is more than five times higher than in the other settings. However, the contribution of adults to the overall force of pneumococcal infection is similar, largely because adults form a much smaller contribution of the overall population in Kilifi. This implies that if high carriage rates in adulthood are sustained as life expectancy increases and the proportion of adults in the community increases, this may lead to a greater contribution of unvaccinated adults to community transmission and challenge PCV herd protection in the future.

Our study comes with a number of limitations. Some of these relate to the model structure. To maintain simplicity and identifiability, we aggregated serotypes into groups and used a model that is not “structurally neutral”, meaning that it tends to promote the long-term coexistence of vaccine and nonvaccine serotypes without an explicit biological mechanism. Moreover, we attributed changes in acquisition probabilities given exposure and duration of carriage to age, while these may in fact be at least in part attributable to exposure. These simplifications were judged acceptable because the model was used to fit steady state data, rather than to make detailed dynamic predictions. Other limitations relate to the representativeness of the available data. While sample size were reasonably large and their associated uncertainty is included in the posterior distributions, only in Kilifi and Nha Trang were both the carriage and contact surveys conducted as a representative sample of the same study population. This mismatch of study populations in Finland and England and Wales could bias our estimates in that carriage information is from adults living in the same household as a child and hence who are likely to be disproportionally more exposed to pneumococci than their childless counterpart. Hence, in these settings, the limited contribution of adults to the infection pressure may still be an over-estimation. Further, in none of the settings were serotyping techniques used that could detect multiple colonisation. However, the role of secondary colonisers for transmission is largely unknown. If secondary, sub-dominant infections play a limited role in transmission, this is unlikely to alter our findings. Further, we assumed that the dual colonisation in our model would be observed as vaccine type carriage; however, when we assumed that such would be attributed to non-vaccine serotype carriage, the results did hardly change because of the relatively low dual colonisation prevalence. The absence of carriage prevalence estimates in adults presented one of the main reasons for exclusion in our study. While there is robust information on adult carriage in Kilifi, only a small number of adults were tested in Nha Trang and adult carriage information in Finland and E&W is restricted to parents and as a result is likely to overestimate carriage rates of the general adult population. Furthermore, we have shown that our results are robust to a number of alternative assumptions, including social rather than physical contacts or alternative carriage clearance rates. For 3 out of 4 countries, the results were also robust to estimating age dependency of transmissibility instead of susceptibility, except for E&W where infants had a much larger role in transmission under this assumption. While in contrast to susceptibility to infection there is currently no evidence for age-dependent differences in transmissibility of pneumococci, from our results alone, we therefore cannot rule out that infants may play a larger role in transmission in E&W. Finally, one of our main outcomes is the quantification of the role of infants in pneumococcal transmission. However, reported information on infant contacts was rare in the studies evaluated. In three of the study sites, only a handful of infants were included in the social contact survey. To account for this and the wider uncertainty in the mixing matrices, we bootstrapped the mixing matrices during the fitting procedure. However, because the surveys were largely filled in by the infant’s mothers, information on contacts during nursery attendance is missing. While childcare attendance rates during the first year of life is likely rather low in Kilifi and Nha Trang [60], we may under-estimate infant contact rates in Finland and E&W.

## Conclusion

In conclusion, we provide a framework for inference of age-stratified population transmission patterns from cross-sectional data on pneumococcal carriage prevalence and social contacts, which adds to the evidence base supporting toddlers and young children as the key drivers of pneumococcal transmission in many settings. We also highlight the potential role of school-aged children and young adults as key vaccine serotype transmitters, particularly in settings with high carriage rates in these age groups. The important contribution that these individuals play may in part explain the reduced herd effects observed in some settings if protection against carriage acquisition from a PCV infant programme wanes to leave these age groups unprotected [61]. Our results support the notion that a reduced dose 1p+1 schedule is likely to sustain herd effects in settings with low residual VT carriage with minimal increased risk for infants before receipt of their booster dose. While our approach allows approximation of transmission on a population level from cross-sectional data, advances in phylogenetic inference may soon allow to substantially increase the specificity of epidemiological linkage by using genomic sequencing, which in turn will allow for better estimates of pneumococcal transmission dynamics.

## Supplementary information

**Additional file 1. **Further technical details on the model.

## Data Availability

All data and code is available at https://github.com/StefanFlasche/Pneumo_Trans_Inf.
