# Supplementary material for: Estimating the contribution of different age strata to vaccine serotype pneumococcal transmission in the pre vaccine era: a modelling study
Source: BMC Med. 2020 Jun 10;18:129. doi: 10.1186/s12916-020-01601-1 (PMC7285529; doi:10.1186/s12916-020-01601-1)

Supplement to
Estimating the contribution of different age strata to vaccine serotype pneumococcal transmission in the pre vaccine era, a modelling study.

By Stefan Flasche^1^, Marc Lipsitch^2^, John Ojal^1^, Amy Pinsent^1^

Further technical details on the model:

Model diagram:


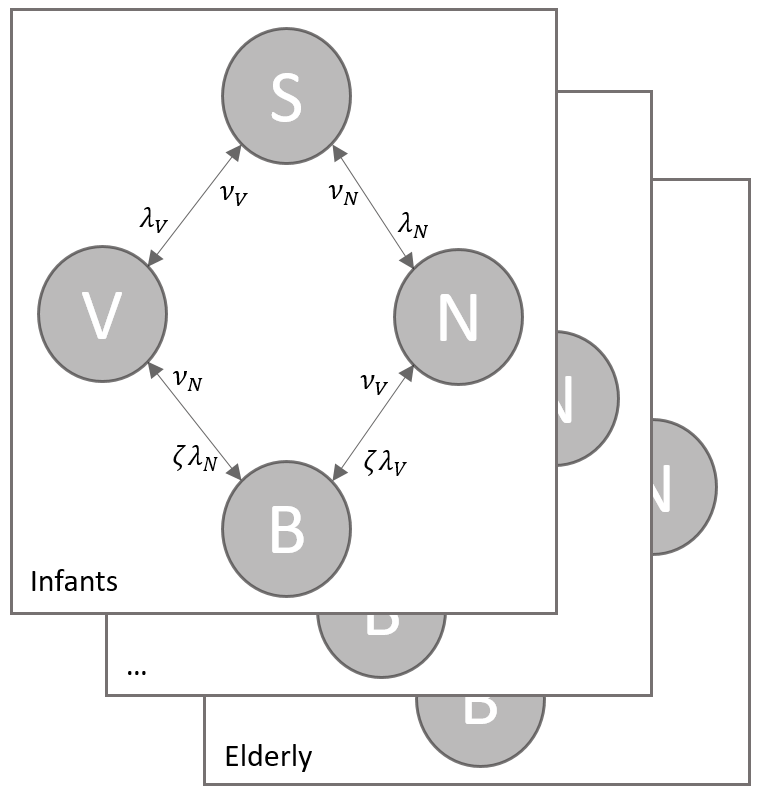


Model equations including aging (subscript $a'$ indicating the respective variable at the next younger age group with $\mu_{a'}=0$ for infants):

$$S^{'} = -\left( \lambda_{V}+\lambda_{N} \right)S+\nu_{V}V+\nu_{N}N-\mu S+\mu_{a'}S_{a^{'}}+\pi(S+V+N+B)$$

$$V^{'}=\lambda_{V}S-\zeta\lambda_{N}V-\nu_{V}V+\nu_{N}B-\mu V+{\mu_{a'}V}_{a'}$$

$$N^{'}=\lambda_{N}S-\zeta\lambda_{V}N-\nu_{N}N+\nu_{V}B-\mu N+\mu_{a^{'}}N_{a'}$$

$$B^{'}=\zeta\left( \lambda_{V}N+\lambda_{N}V \right)-\left( \nu_{V}+\nu_{N} \right)B-\mu B+\mu_{a^{'}}B_{a'}$$

$$\lambda_{V}=\beta_{V} \left( V+B \right)$$

$$\lambda_{N}=\beta_{N} \left( N+B \right)$$

Model parameters:

| Parameter | interpretation | Value |
| --- | --- | --- |
| $\beta_{k,i,j}$ | Effective contact rate for a specific individual in age group j with one in age group I for serotype group $k\in\{VT, NVT\}$ | $=t_{k,j}c_{i,j}\theta_{k,i}$ |
| $c_{i,j}$ | Average number of physical contacts for a specific individual in age group j with one in age group i | From contact surveys in the settings (see table 1) |
| $t_{k,j}$ | Infectivity of an infected individual in age group j for serotype group k | 1 for the base case |
| $\theta_{k,i}$ | Susceptibility of an individual in age group i to infection if exposed to serotype group k | Estimated for 4 age groups |
| $\nu$ | Clearance rate for infections | From country specific estimates (see table 1) |
| $\mu$ | Aging rate | 1 / number of years in the respective age band |
| $\pi$ | Birth rate | $=\mu$ for the oldest age group and 0 otherwise |

Figure S1: model fit to observed carriage prevalence. Gray shows the central 95% of the posterior distribution, and the dots (error bars) show the observations and associated uncertainty


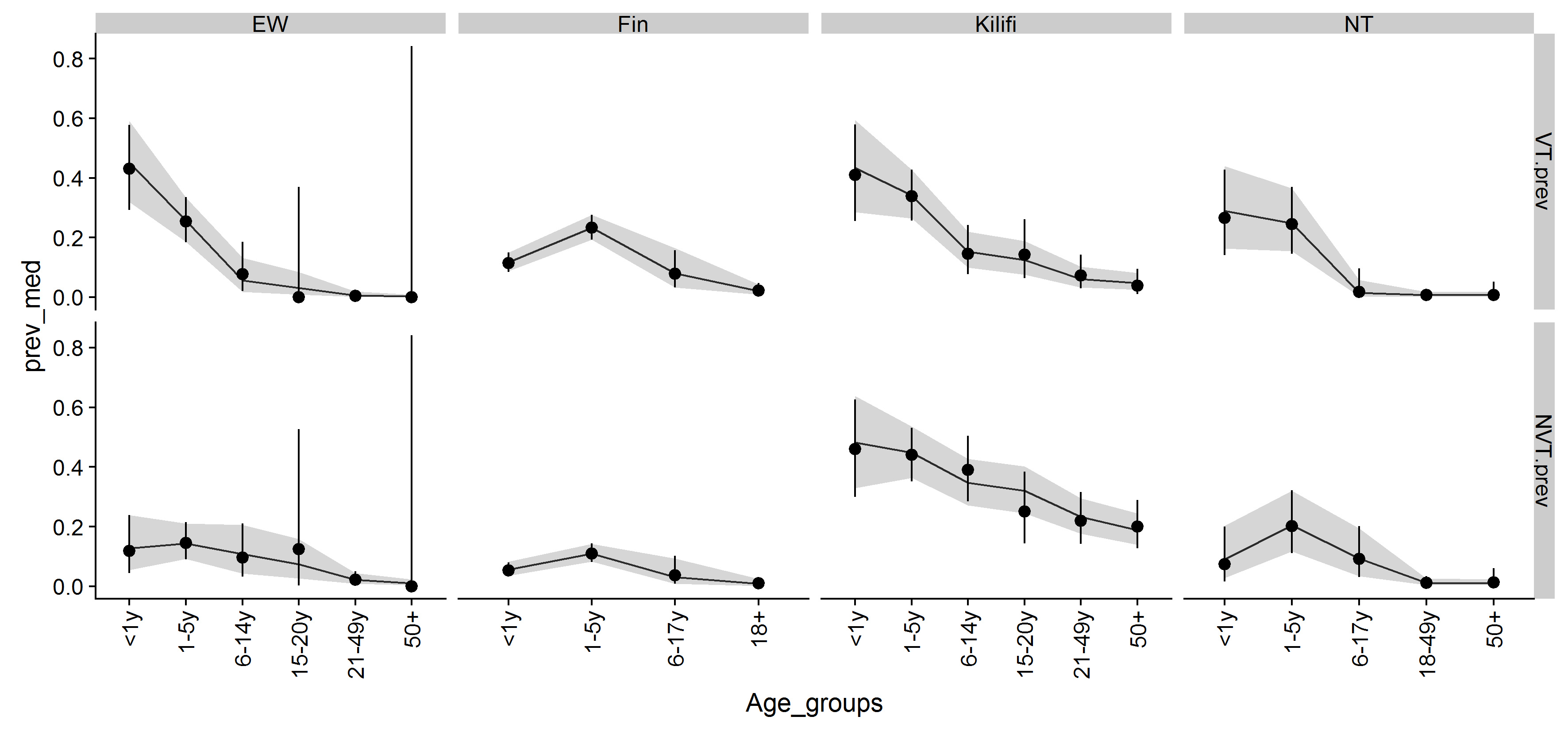


Figures S2a-c: correlation plots of the posterior samples for the fitted parameters for a) England and Wales, b) Finland, c) Kilifi and d) Nha Trang


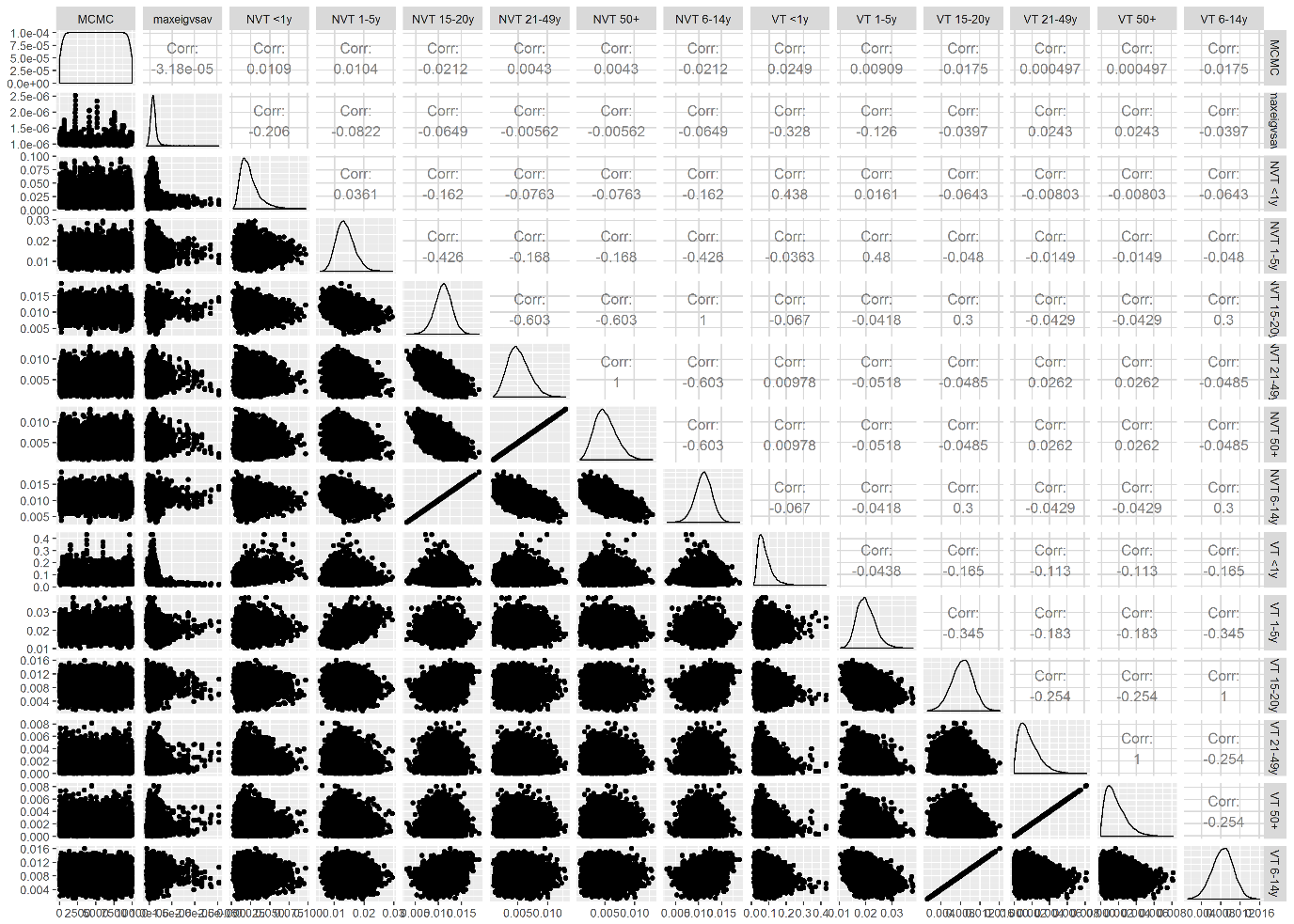


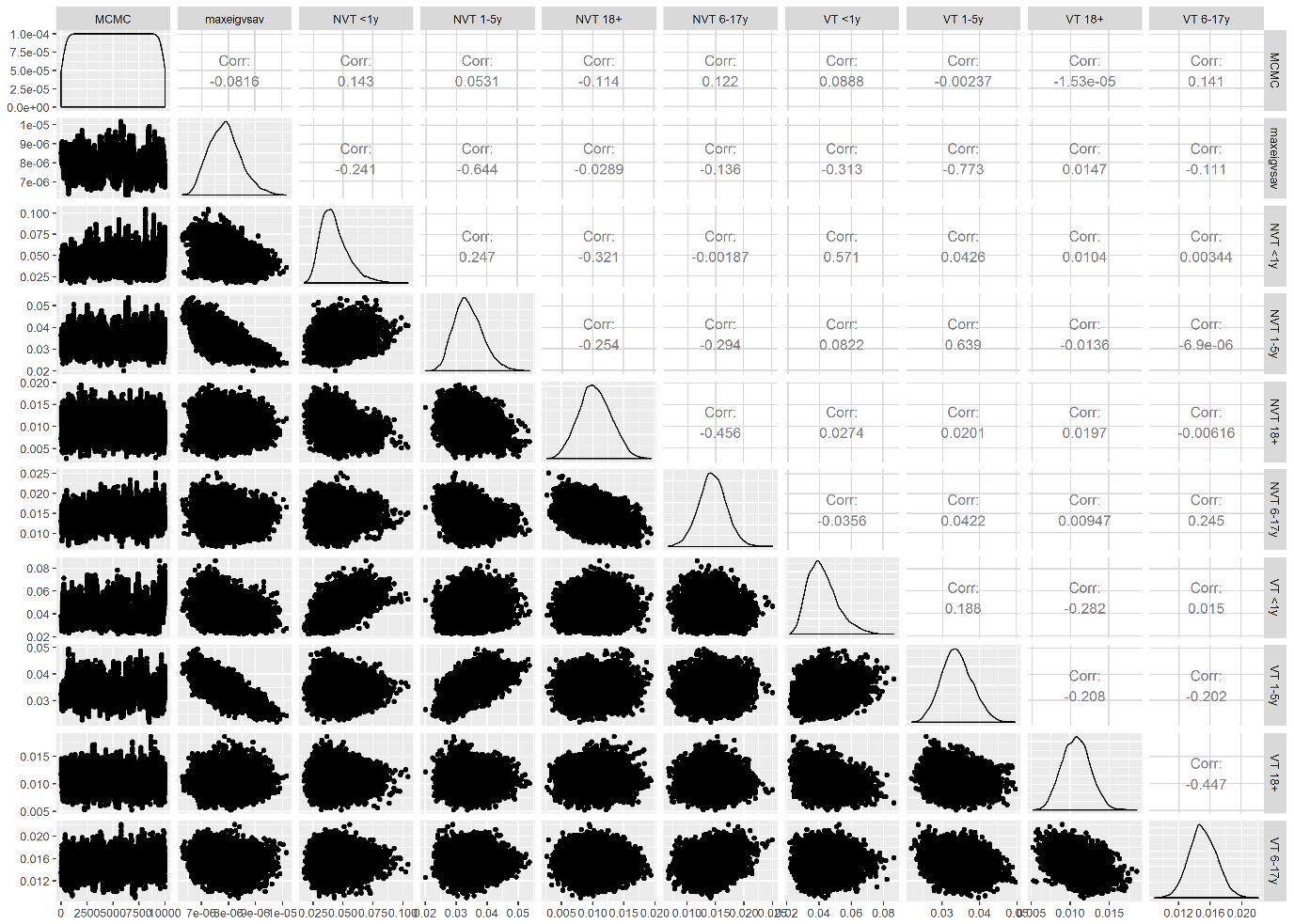


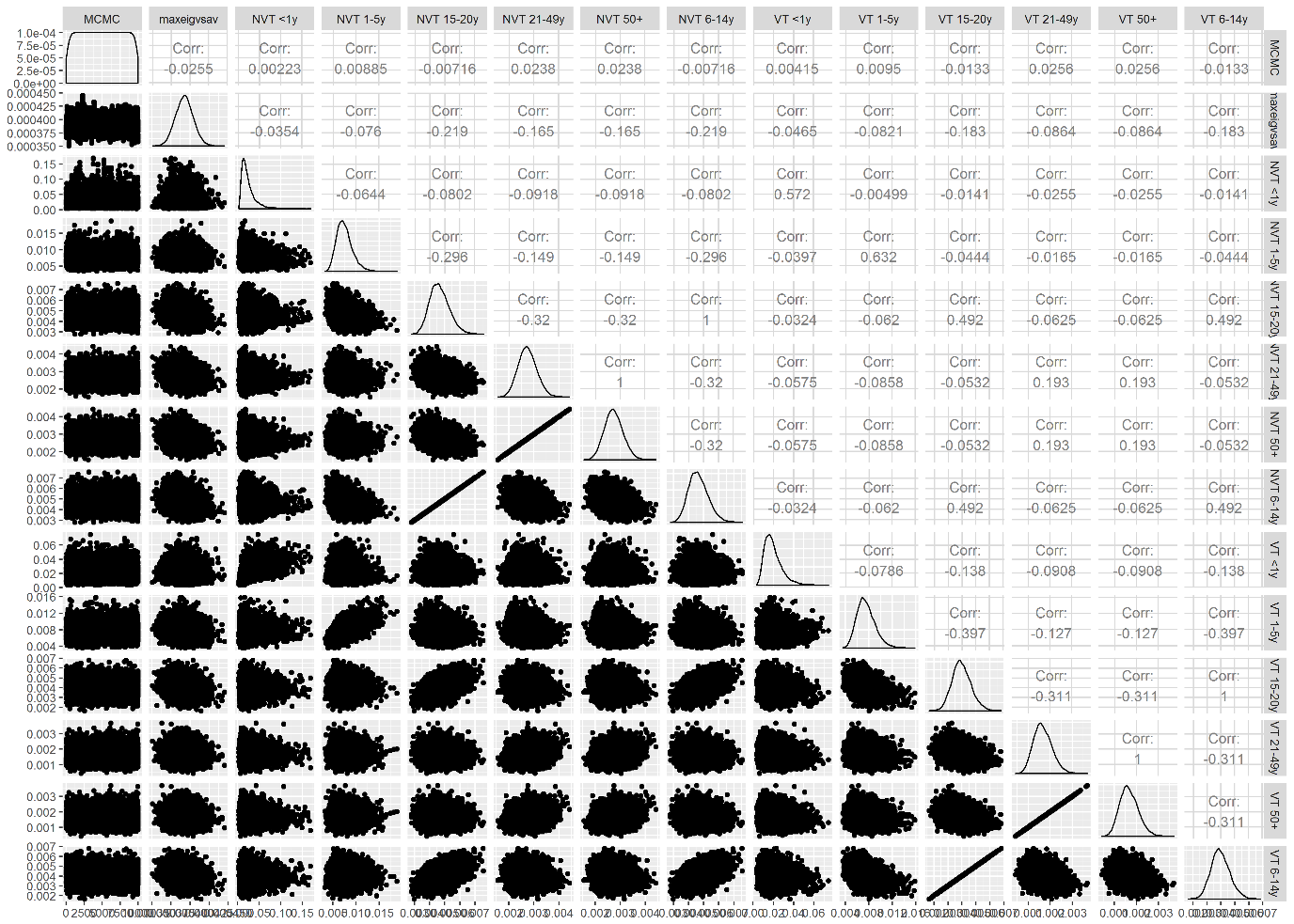


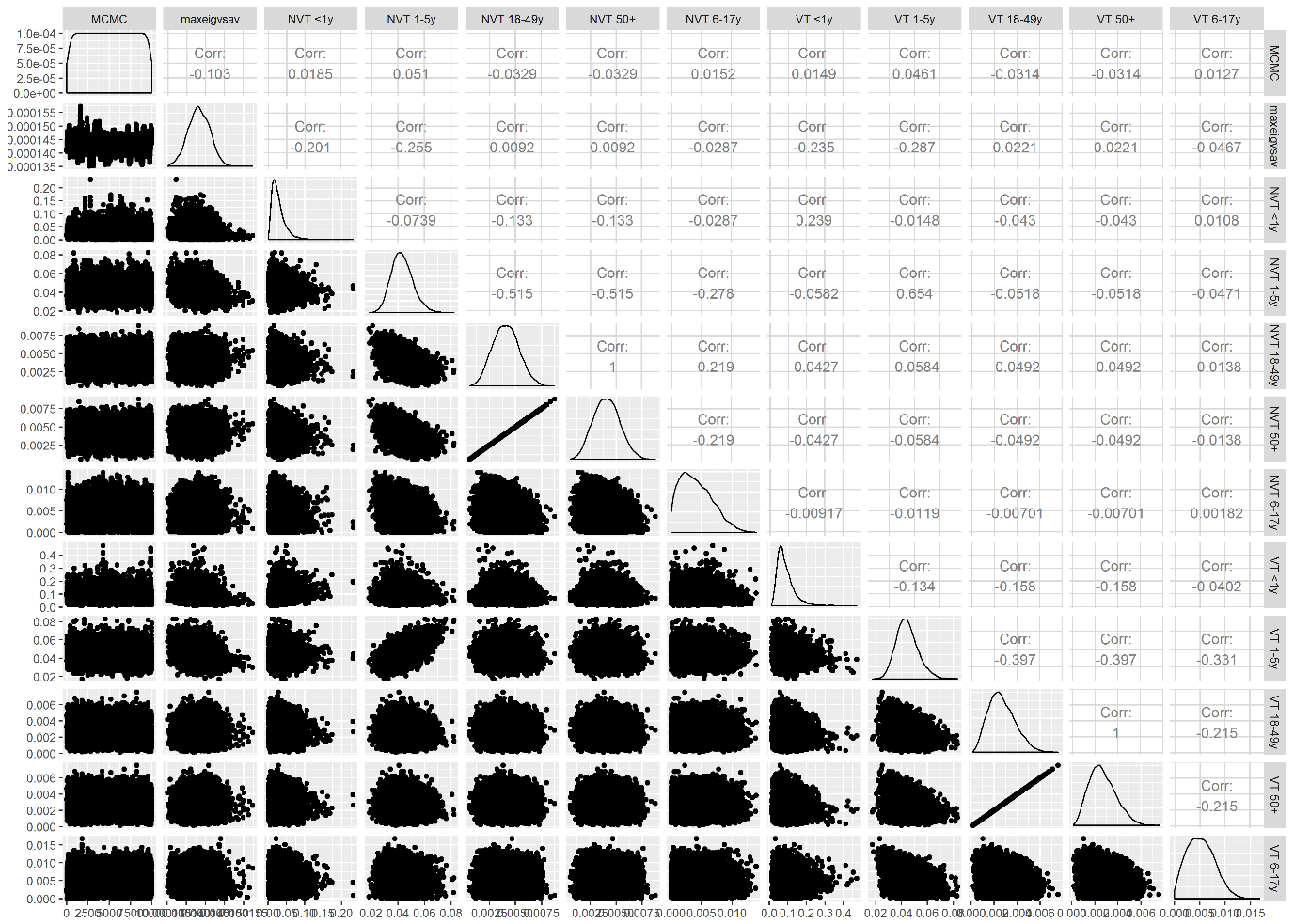


Figure S3: model fit to observed carriage prevalence if fitting transmissibility instead of susceptibility


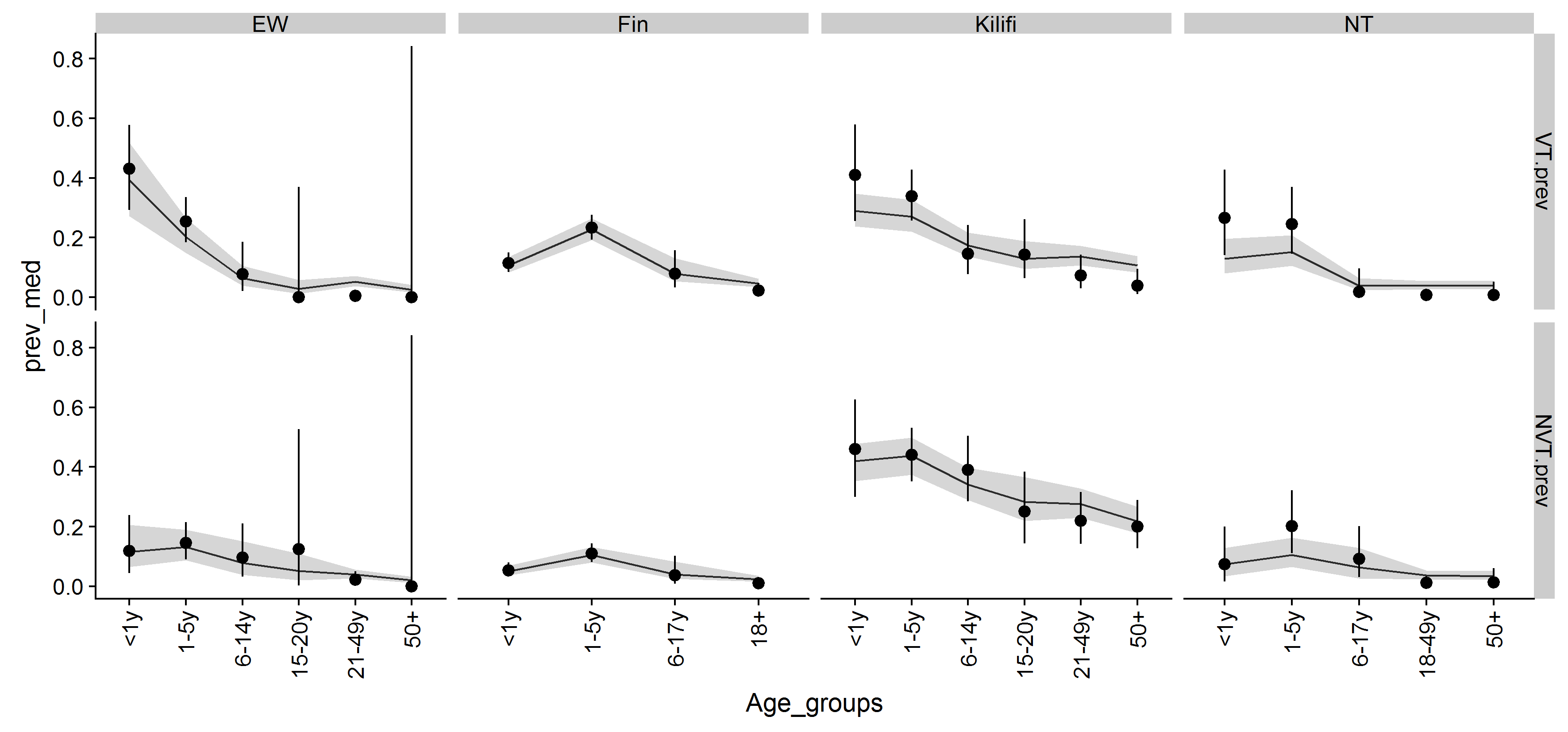


Figure S4: attribution of all transmission events a) to all population b) to infants if fitting transmissibility instead of susceptibility

a)
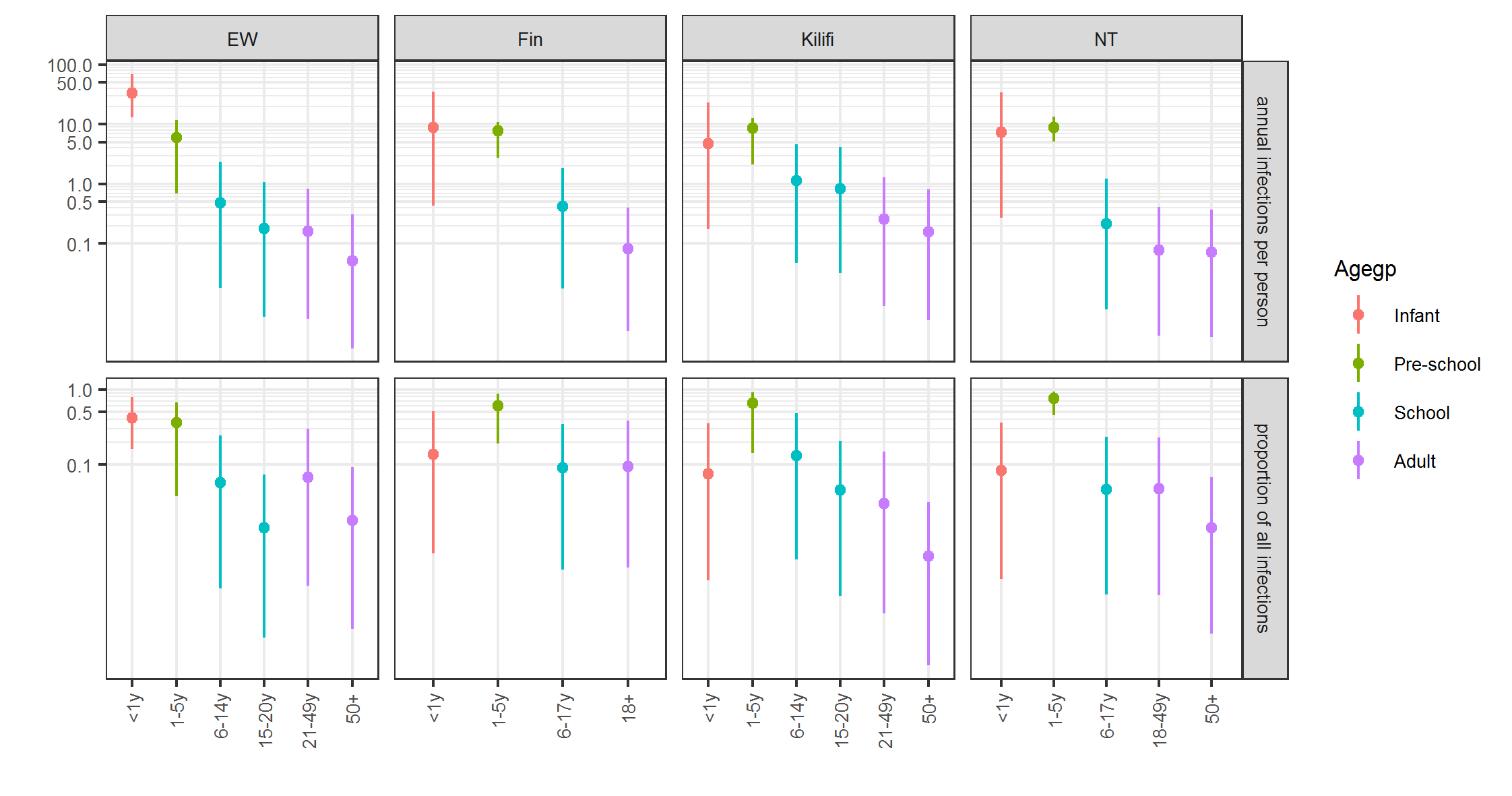


b)
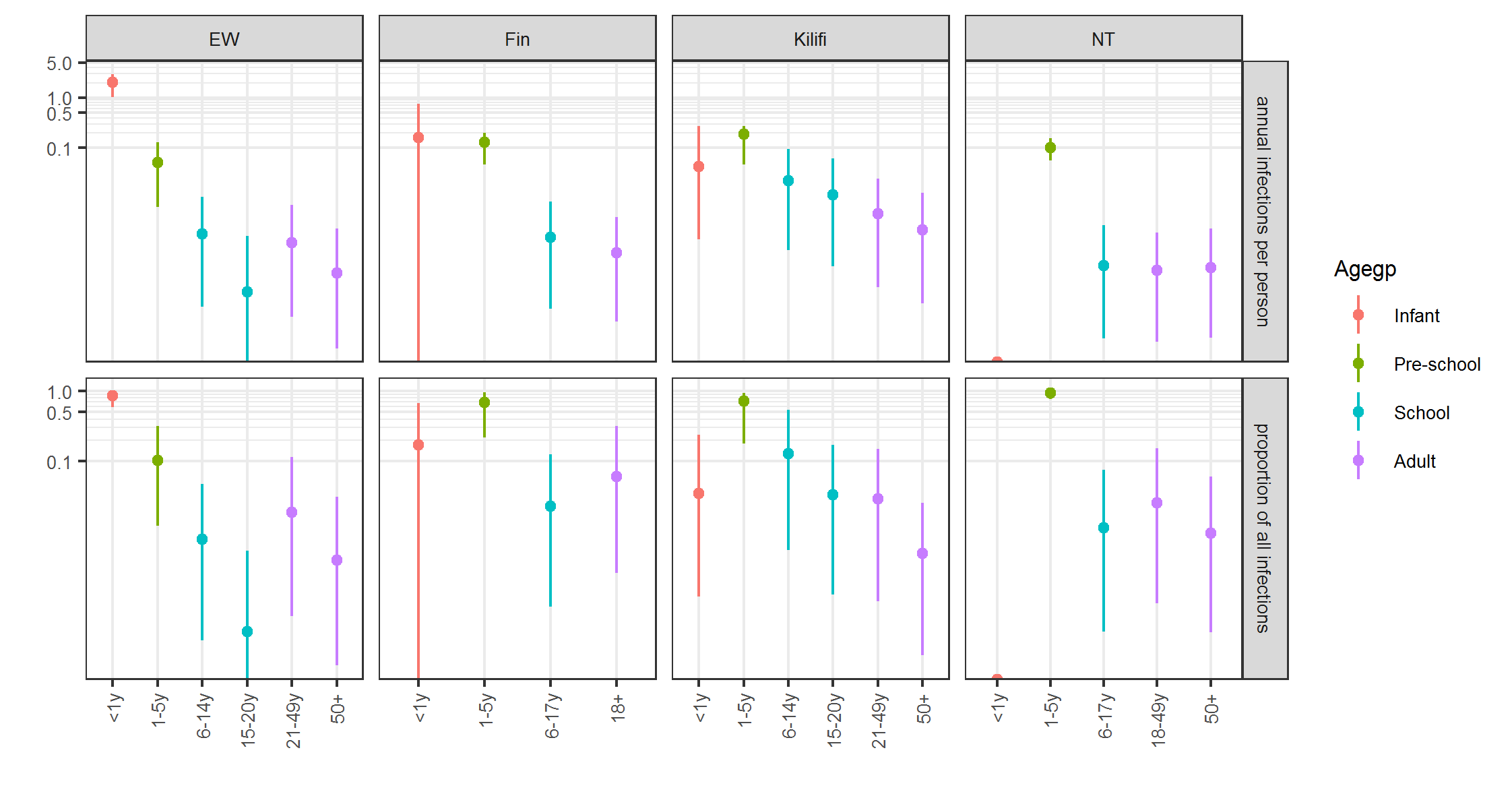


Figure S5: attribution of transmission events if fitting transmissibility instead of susceptibility


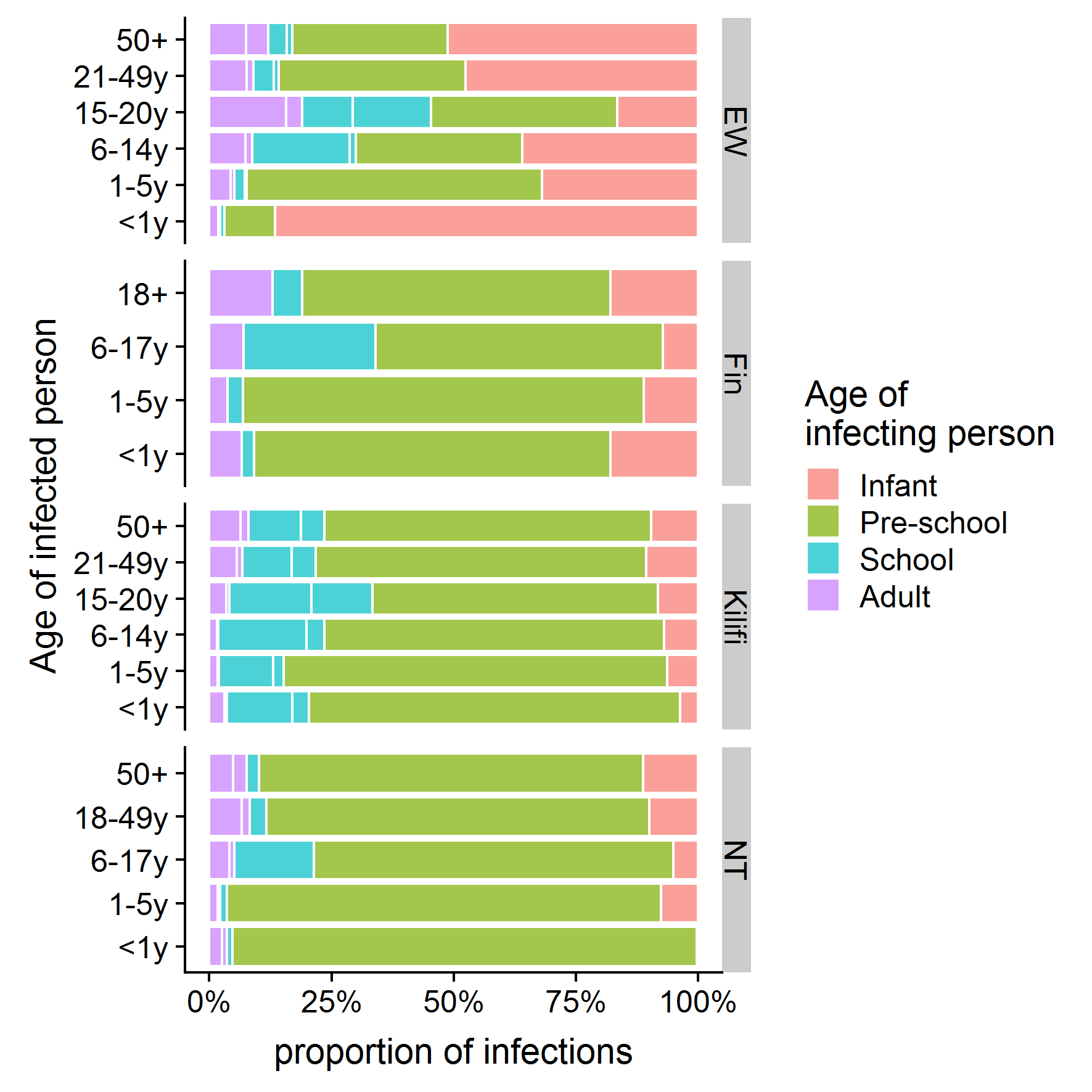


Figure S6: attribution of transmission events for Nha Trang if assuming Kilifi-like clearance rates instead of EW-like clearance rates in Nha Trang
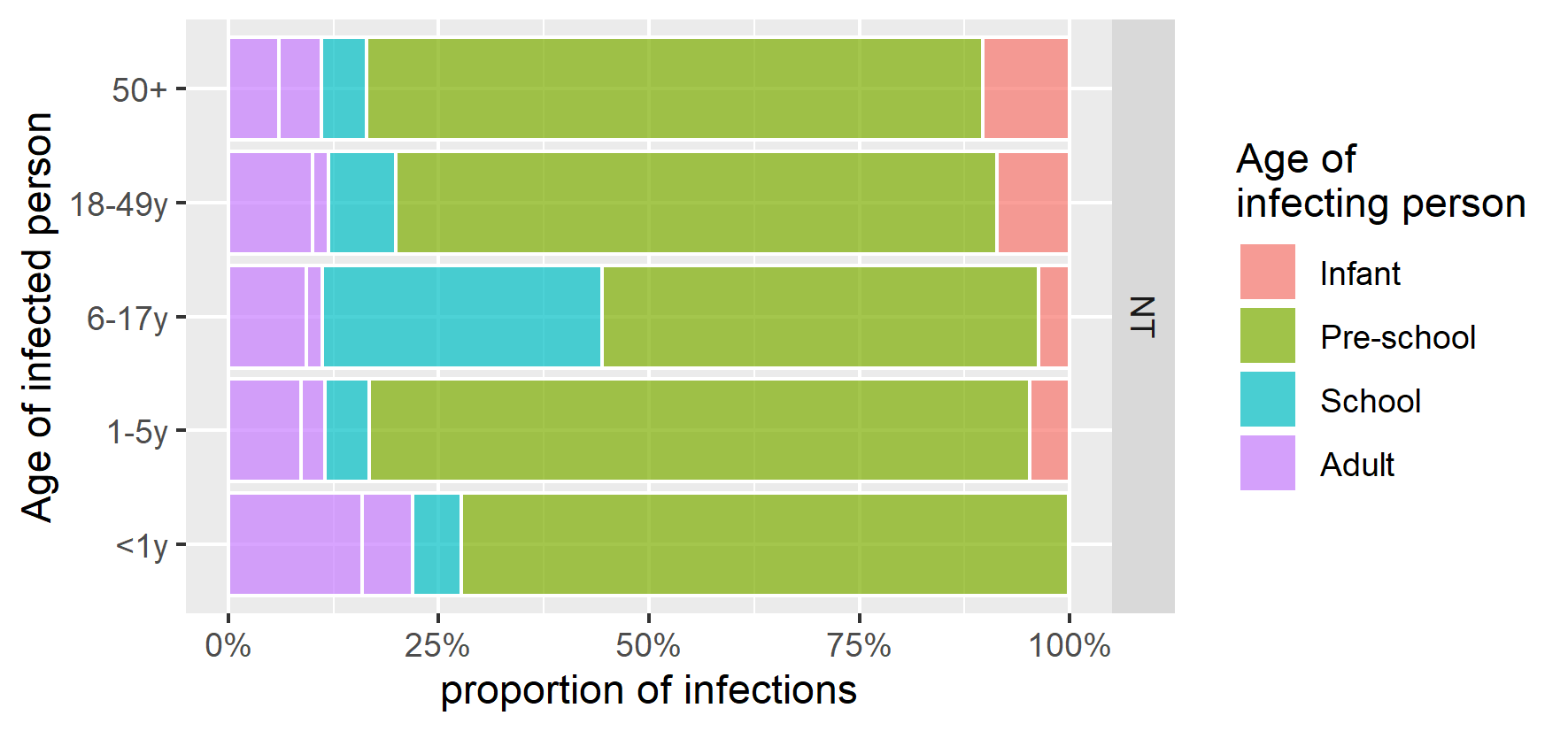


Figure S7: attribution of transmission events for England and Wales, Finland and Nha Trang if assuming that all social contacts rather than only physical contacts are relevant to the transmsision of pneumococi


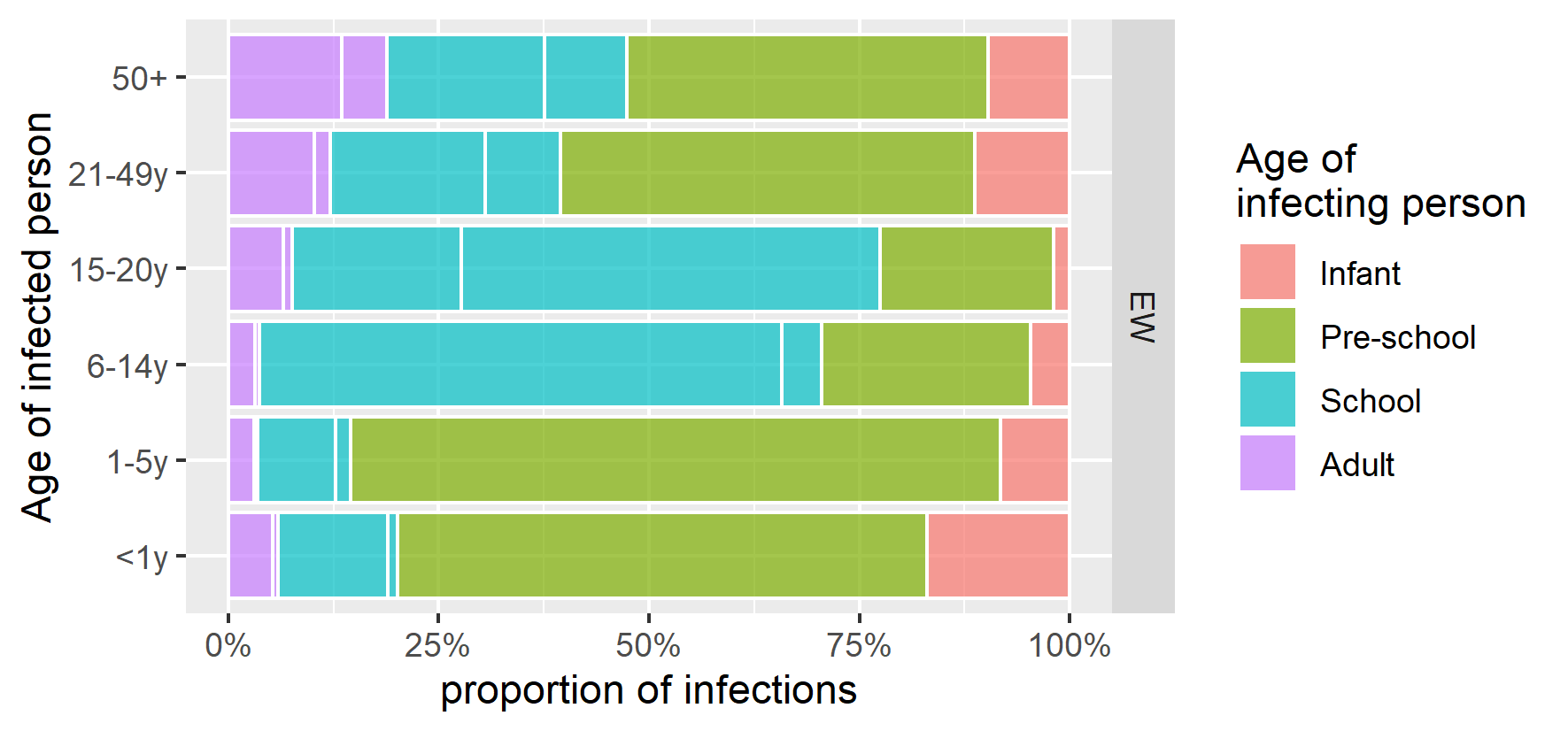


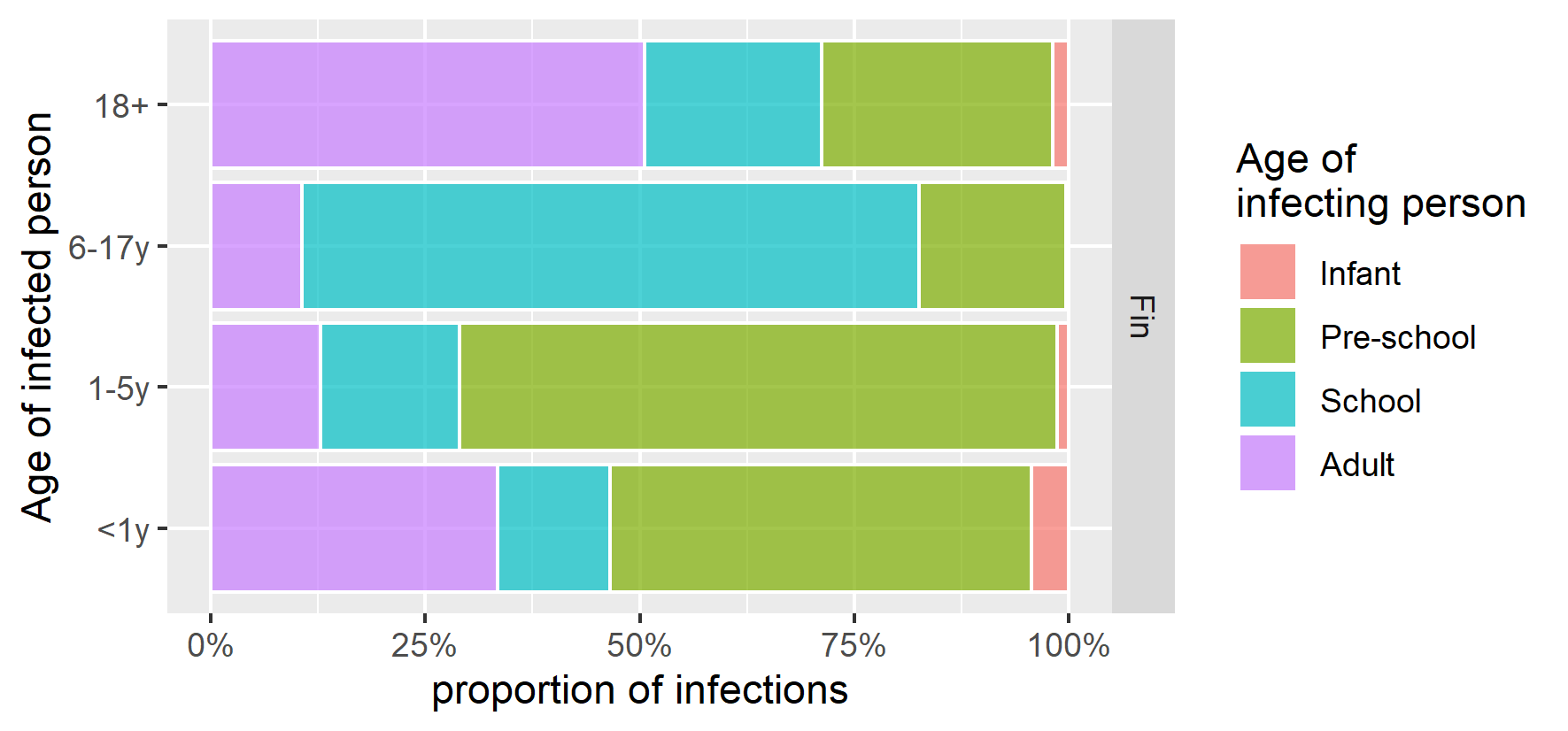


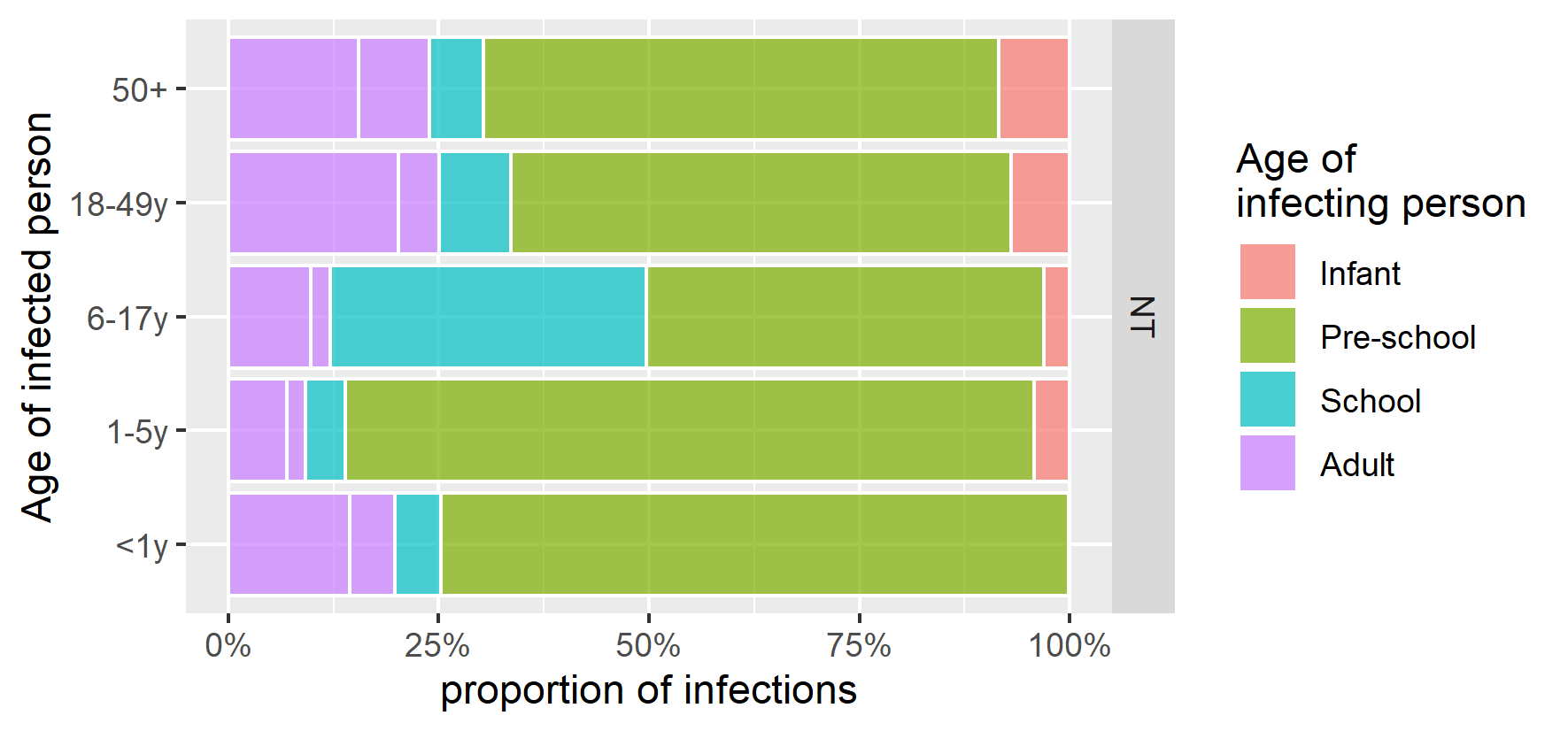


Figure S8: attribution of transmission events for England and Wales if assuming less serotype competition, ie c=0.3 instead of c=0.1.


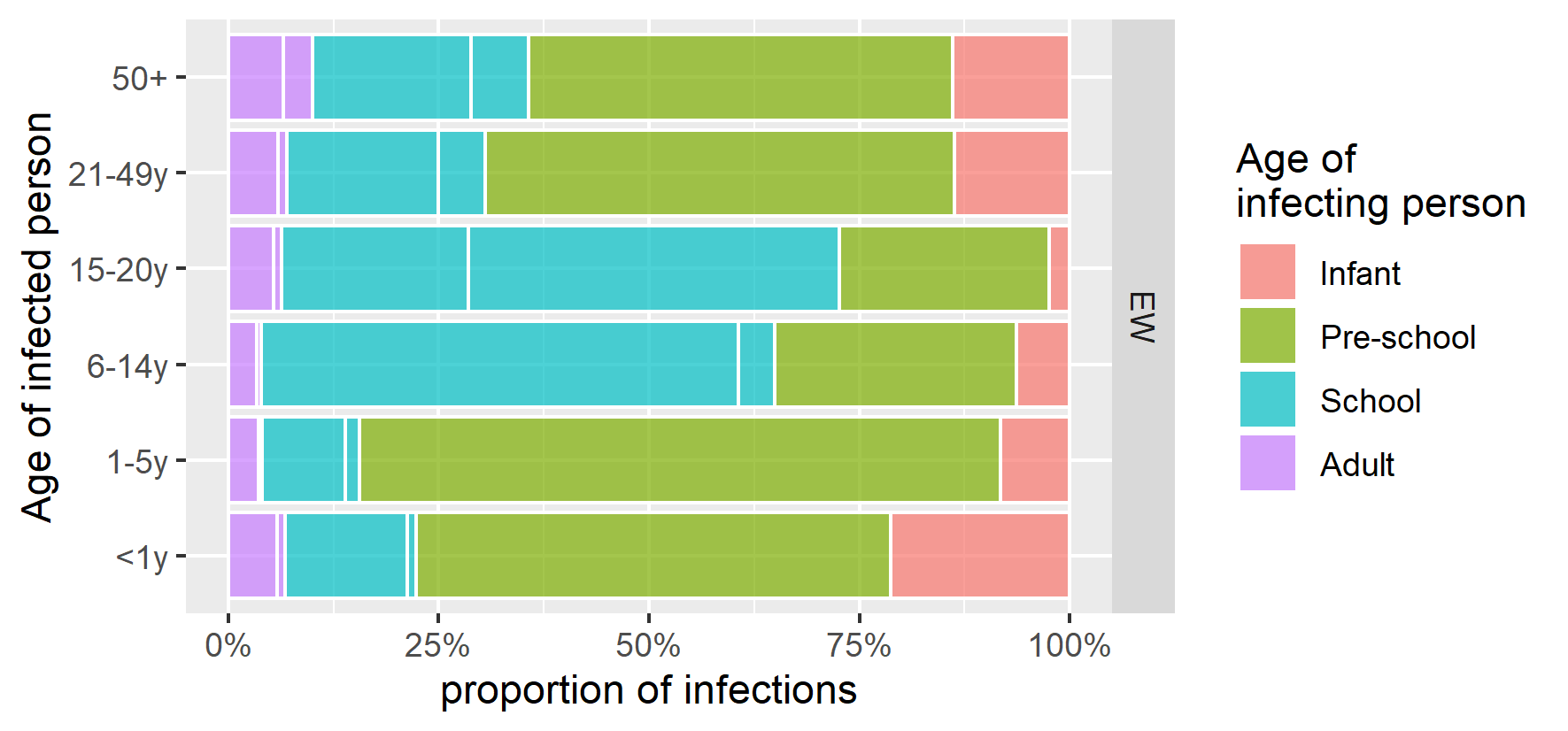

Supplement: Supplementary file 1 — Additional file 1. Further technical details on the model. [file 12916_2020_1601_MOESM1_ESM.docx]
